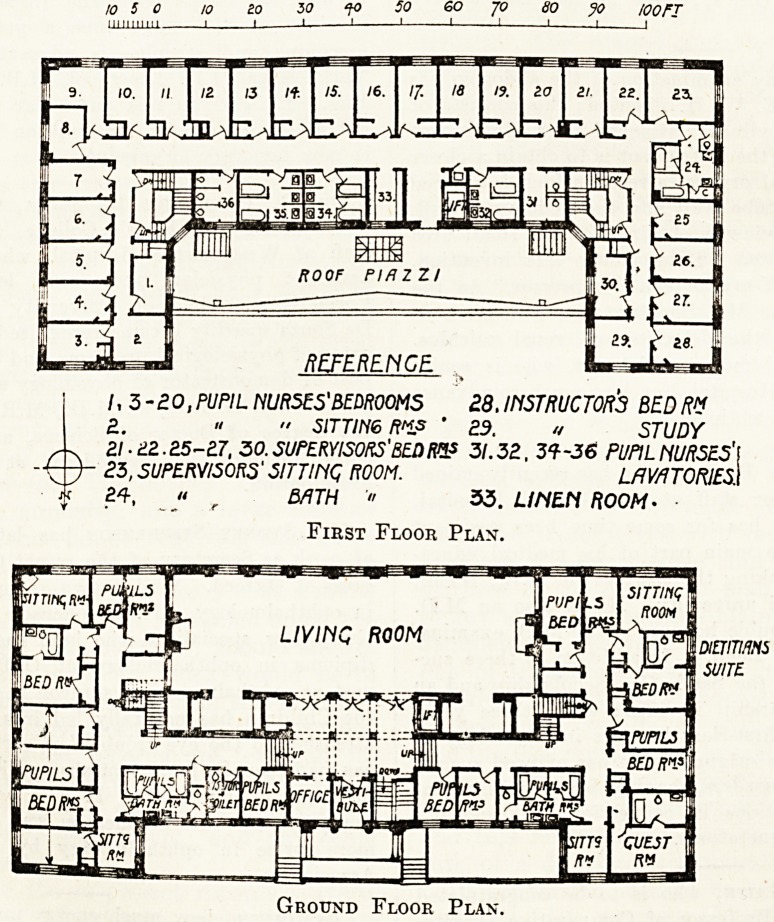# The Nurses' Home at the Massachusetts General Hospital

**Published:** 1914-07-18

**Authors:** 


### HOSPITAL ARCHITECTURE AND CONSTRUCTION.

### The Nurses' Home at the Massachusetts General Hospital.

The newly erected Nurses' Home is designed as an auxiliary to the already existing Thager building, which has accommodation for 113 women. It is separated from the hospital grounds by a public street, and connection beween the two is by way of a subway under the street.

The new building contains accommodation for 101 nurses in separate rooms, with two suites of bedroom, sitting-room, and bathroom—one for the superintendent of nurses, the other for the dietitian.

In the lowest or half-basement storey there is a large classroom, a recreation-room, sewing-room, trunk-room, and a small laundry, with other rooms, to which no purpose is at present assigned. The classroom is about 63 feet long by about 20 to 24 feet wide, with a platform at one end. At the other end is a door leading into what apparently is a pantry, which rather suggests that the room is intended to be used for some sort of meal.

On the ground floor the main entrance from the street is on the north. A vestibule with double doors gives access to a spacious entrance hall divided up by columns and pilasters into bays. Three wide pairs of doors give access to the living-room, a room about 75 feet long by 25 feet wide, lighted by seven windows on the south wall. provided with a fireplace at each end and two recesses on the north side. Adequate height is obtained for the living-room by keeping the vestibule floor some 3 feet below the general ground-floor level. This necessitates two flights of six steps each from the vestibule to the corridor running east and west.

The remainder of the ground floor is occupied by bedrooms for pupils, the two suites referred to above, a guest-room with sitting-room and bathroom attached, four bathrooms for pupils, and a visitors' toilet-room.

The first, second, and third floors are identical, and contain bedrooms, bathrooms, and w.c.'s, with a central linen-room on each floor.

First Floor Plan.

Ground Floor Plan.

An electric lift, fitted with automatic push-button control, ascends from the basement to the top floor.

The building is made of fire-resisting materials throughout; the floors are of reinforced concrete, and, except in the living-room, vestibule, and the bathrooms, are covered with linoleum cemented to the surface. The living-room is floored with quartered oak, the vestibule with Italian marble, and the bathrooms and toilet-rooms with terrazzo.

The architects were Messrs. Sheply Rutan and Coolidge, of Boston, U.S.A.

## Figures and Tables

**Figure f1:**